# Constructing and evaluating the physical literacy index for college students in China: a new insight and perspective

**DOI:** 10.3389/fpubh.2025.1612356

**Published:** 2025-06-23

**Authors:** Hongbiao Wang, Zhongsu Piao, Zhiguang Ji, Leichao Liang, Qianhong Chen, Ruoyu Yang, Ming Cai, Liyan Wang

**Affiliations:** ^1^Department of Physical Education, Shanghai University of Medicine & Health Science, Shanghai, China; ^2^Physical Education College, Jilin University, Changchun, China; ^3^College of Rehabilitation, Shanghai University of Medicine & Health Science, Shanghai, China

**Keywords:** college students, physical literacy index, assessment, principal component analysis, health promotion

## Abstract

**Background:**

College students today face significant challenges related to physical literacy, including declining fitness levels and low health awareness. Constructing a scientifically robust index to assess physical literacy in this demographic is essential.

**Methods:**

We distributed 800 questionnaires via simple random sampling across eight universities, ultimately obtaining 706 valid responses from first- and second-year undergraduate students in Shanghai. Various components of physical literacy—including exercise motivation, attitude, commitment, confidence, body appreciation, willpower, physical activity, and fitness—were measured using established tools. Data analysis was conducted using SPSS 26.0, covering descriptive statistics, reliability and validity assessments, correlation analysis, and difference analysis.

**Results:**

The Physical Literacy Index (PLI) for Chinese college students was developed through principal component analysis. The weights for the components were as follows: motivation for sports (0.171), attitude toward sports (0.147), physical fitness (0.163), commitment to sports (0.130), physical activity level (0.105), appreciation of the body (0.122), confidence in sports (0.091), and willpower in sports (0.070). The PLI showed a significant negative correlation with screen time (correlation coefficient of −0.257) and strong positive correlations with other variables, with the lowest correlation coefficient at 0.594, indicating effective calibration. The overall physical literacy level among Chinese college students is concerningly low, with no significant differences found across genders, ages, or regions of origin (*p* < 0.05). Despite a relatively high pass rate for physical fitness tests (64.16%), only 1.42% achieved excellent grades, and just 21.67% scored good.

**Conclusion:**

The physical literacy index of Chinese college students constructed in this study is scientific and valid, but the current situation regarding college students’ physical literacy is worrying. In the future, physical education and health promotion should be emphasized and targeted measures should be taken to improve the physical literacy of college students.

## 1 Introduction

College students, as an important talent pool for national development, have received considerable attention for improving their overall quality. As an important part of college students’ comprehensive development, physical literacy (PL) has a critical impact on their academic performance, professional development, and quality of life (1). With the rapid development of science and technology and the change of lifestyle, college students ‘physical quality is facing many challenges. For example, the habit of sitting for a long time, excessive dependence on electronic products and unhealthy eating habits lead to the decline of college students’ physical quality, and problems such as obesity and myopia become more serious (2). In addition, some college students have a biased understanding of physical exercise, lack active participation in sports activities, low sports participation, and poor health awareness (3). Therefore, it is of great practical importance and urgency to construct a scientific and reasonable PL index for college students and to make an accurate assessment of their PL.

The concept of PL originated in the late 19th century; for example, in 1884, Captain Edward Maquire of the United States first used the term “PL” in his professional notes and described the characteristics of physical activity (PA) in indigenous cultures (4). Since then, the concept of PL has gained attention and has evolved throughout the 20th century. Margaret Whitehead first introduced the modern concept of PL in 1993 and has further enriched and refined the definition in subsequent research (5). She defined PL as “the motivation, confidence, physical competence, knowledge, and understanding to maintain PA throughout the life course” and emphasized the importance of PL as an essential part of a person’s life. The life course emphasizes that PL is a lifelong process that involves multiple aspects of an individual’s motivation, self-confidence, physical competence, knowledge and understanding (6).

PL is a complex and multidimensional concept that encompasses multiple aspects of a person’s motivation, self-confidence, physical abilities, knowledge, and understanding (3). Its core structure typically encompasses several key elements. First, there are physical elements, such as fundamental movement skills, physical competence, and physical fitness. Second, psychological elements, including motivation and self - confidence, are integral. Third, cognitive elements play a crucial role, which involve knowledge, understanding, attitudes, and appreciation of PA. Fourth, behavioral elements consist of physical activity and sedentary behavior. Fifth, social elements, mainly manifested as interaction with others, are also part of the structure. Additionally, the ability of reading and interacting with the environment is included. Finally, a focus on the lifelong journey is an essential characteristic of its core structure (1). To effectively understand an individual’s level of physical literacy (PL), appropriate measurement and assessment are essential. These measurement methods include qualitative (e.g., interviews, questionnaires, observations, reflective diaries, focus groups), quantitative (e.g., physiological indicators, motor skill tests, psychometrics), and integrative approaches (e.g., mixed-methods studies, multidimensional assessments) (7–16). However, PL measurement also faces many challenges, such as conceptual complexity, limitations of measurement methods, influence of individual differences, and role of environmental factors (3, 15, 17, 18).

In China, there are still issues with research and testing tools for college students’ PL. Several key challenges remain in the measurement and assessment of physical literacy (PL). These include the absence of a unified measurement framework, the need for more robust and validated measurement methods, insufficient interdisciplinary collaboration, limited practicality in real-world settings, and a lack of strong international cooperation and knowledge-sharing (10, 19–22). In addition, Chinese PL research still needs to be thoroughly investigated in terms of its theoretical foundations, research limitations, monitoring and acceptance, and improvement mechanisms and means (23).

Moreover, while the exploration of Chinese PL research’s theoretical and practical aspects remains an ongoing task, the rich philosophical heritage of China offers valuable resources. Thus, concepts such as cognition, emotion, will, and action from traditional Chinese philosophy, when applied to the construction of PL, provide new perspectives and methods for the comprehensive assessment and improvement of individuals’ PL (7). In this study, the test content and structure of PL are constructed from the four aspects of “cognition, emotion, will and action,” including sports cognition (training motivation, training attitude), sports emotions (training commitment, training confidence), aspects of sports will (willingness to exercise, willpower), sporting behavior (PA, physical fitness) (24). By comprehensively examining these aspects, an individual’s PL level can be assessed more comprehensively and in depth, providing strong support for physical education and health promotion (25).

In summary, establishing a scientific and reasonable PL index for college students and accurately evaluating it are of great importance to improve the PL of college students and promote their all-round development. At the same time, it helps promote the reform of physical education in colleges and universities and cultivate more high-quality talent for the country. This study constructs the physical fitness and literacy index of college students, which not only helps to precisely promote the reform of physical education in colleges and universities and rationally allocate educational resources, but also provides an objective basis for college students’ self-awareness, which is of great significance for promoting the all-round development of college students’ physical and mental health.

## 2 Methods

### 2.1 Ethics approval and consent to participate

The study was carried out following the principles of the Declaration of Helsinki. The Research Ethics Committee of Shanghai University of Medicine and Health Sciences approved the study. All participants gave informed consent prior to the survey.

### 2.2 Participants

The respondents in this study were current freshmen and second-year students enrolled in higher education institutions in Shanghai. Using simple random sampling, 800 questionnaires were distributed to eight colleges and universities (two 985, two 211, and four urban colleges and universities). We selected two 985, two 211, and four urban colleges in Shanghai through random sampling from each category to represent different levels and educational focuses of local higher education, covering diverse student profiles and experiences. To gain access for questionnaire distribution, we first established contact with universities’ administrative departments, presenting our research proposal and emphasizing its significance. Our research had already obtained ethical approval from our institution’s review board, which served as proof of its ethical compliance when approaching universities. We ensured students’ voluntary participation by providing detailed informed consent forms, clearly stating the study’s purpose, data usage, and confidentiality, and distributing questionnaires only after getting their consent. After screening the recovered questionnaires, 94 invalid questionnaires were excluded according to the “omissions or incorrect answers” criterion. The reverse-scoring question as well as the same selection in more than one question were invalid questionnaires, and 706 valid questionnaires were finally obtained (88, 25%).

### 2.3 Measures

#### 2.3.1 Exercise motivation measurement

When measuring exercise motivation, the simplified version for measuring exercise motivation compiled by Prof. Shan-ping Chen (26) was adopted, which is a 15-point scale. The metric uses a 5-point Likert scale, with higher values indicating higher and stronger motivation. In the internal consistency coefficient (Cronbach’s *α* coefficient) of the scale, the reliability coefficient of the fun motivation dimension was 0.725, that of the ability motivation dimension was 0.733, and that of the appearance motivation dimension was 0.806. The reliability coefficient of the “health motivation” dimension is 0.750 and the reliability coefficient of the “social motivation” dimension was 0.899. All subscales had a very good score, indicating that the scale had good internal consistency. Exercise motivation is the total or average score of the five subdimensions added together. The higher the total score, the stronger was the motivation to exercise. The internal consistency coefficients for the five dimensions ranged from 0.793 to 0.848 (26).

#### 2.3.2 Exercise attitude measurement

The exercise attitude measurement was adopted from the “Physical Exercise Perseverance Scale” (27) developed by Shen Wang. This scale includes 14 items that are “effort commitment” [e.g., (5–9); I will do my best to practice new skills], emotional experiences [(10–14), for example, I often feel positive after training] and behavioral habits [(1–4), e.g., it has been at least 6 months since I started PA] Dimensions. Responses were rated on a 5-point scale ranging from “strongly disagree” to “strongly agree” on a scale of 1 to 5. The higher the total score, the higher the adherence to exercise behavior. The Cronbach’s *α* coefficient of the scale was 0.958, which is greater than 0.7, indicating that the internal consistency of the questionnaire is good and has good reliability and validity indicators. The reliability coefficients of the subscales were 0.900, 0.809, and 0.804 (27).

#### 2.3.3 Exercise commitment measurement

To measure exercise commitment, the Physical Exercise Commitment Scale developed by Prof. Shan-ping Chen was used (26), which consists of 8 items on a 5-point Likert scale ranging from “strongly agree” to “strongly disagree” is enough on a scale of 5–1. Questions 1–4 were used to measure exercise engagement. The higher the score, the stronger was the exercise. This reflects the individual’s psychological commitment to exercise and behavioral intention to adhere to exercise. Questions 5 to 8 on the scale were used to measure actual exercise adherence behavior. The higher the score, the stronger the behavioral intention, which reflects the individual’s psychological commitment to physical exercise and behavioral intention to adhere to physical exercise at the behavioral level. The discriminant and convergent validity of these two variables were examined using confirmatory factor analysis (CFA) in LISREL. The chi-square value was 301.30, df = 19, GFI = 0.96, AGFI = 0.93, NFI = 0.98, and CFI = 0.98, indicating that the data of the two dimensions were well fitted, suggesting that the discriminant validity of the scale was relatively satisfactory. This also proves that the two measurement models have a good external validity. In this study, the alpha reliability coefficients of the subscales were 0.839 and 0.921 (26).

#### 2.3.4 Exercise confidence measurement

The exercise confidence measure used a simplified version of the exercise self-efficacy scale developed by Chen (26) and was rated on a 5-point Likert scale. The scale describes six types of situations that can make PA difficult, and subjects are asked to determine their level of confidence in maintaining a regular PA routine under these circumstances using the following options: not at all likely, less likely, fairly more likely, more likely, and more capable. To understand whether the simplified version of the scale still exists in the possible classification, an exploratory factor analysis was carried out to measure the subject; the specific method of principal component analysis and the maximum variance (Varumax) method of orthogonal line rotation (orthogonal rotation), the factor analysis of the KMO (Kaiser-Meyer-Olkin Measure of Sampling Adequacy) was 0.831, and the chi-square value of Bartlett’s Test of Sphericity was 2771.27 (with 21 degrees of freedom). This reached a significance level of 0.001, indicating that it was suitable for the factor analysis. However, there was only one factor with an eigenvalue greater than 1, indicating that there was only one common factor, and there was no second-order factor structure for the six simplified questions. The coefficient of the scale is 0.839. In this study, the reliability coefficient of the scale was 0.790 (26).

#### 2.3.5 Body appreciation measurement

Body appreciation was measured using the Body Appreciation Scale-2 (BAS-2), a Chinese version developed by Ma et al. (28), a unidimensional scale that assesses the degree to which an individual’s overall acceptance and appreciation of their own body is more stable and consistent. Higher scores on the scale indicate greater appreciation for one’s body shape. The scale consists of 10 items and is rated on a 5-point scale, where 1 means “never” and 5 means “always.” The internal consistency coefficient of the scale was 0.931, and Cronbach’s *α* coefficient for the scale in this study was 0.89 (28).

#### 2.3.6 Exercise willpower measurement

PA perseverance was measured using the Brief Grit Scale revised by Wang (29). The scale contains two dimensions, h. Interest rate stability factor contains questions 1, 3, 5, and 6, and effort persistence factor contains questions 2, 4, 7, and 8. It consists of 8 items, such as “Frustration does not discourage me” and “I am a hard worker.” A 5-point Likert scale was used, with questions 1, 3, 5, and 6 being reverse-scored. Ratings ranged from 1 to 5 from “and does not conform” to “and conforms.” In this study, Cronbach’s *α* coefficients for the two subscales were 0.768 and 0.771, respectively, with good reliability (29).

#### 2.3.7 PA measurement

The PA Rating Scale developed by the Japanese scholar Koyo Hashimoto and revised by our scholar, Liang, was used to assess the PA level of students (30). The scale assesses activity levels by asking students about the intensity, frequency, and duration of their participation in physical activity over the past month. Intensity was categorized as low, medium, or high, and the scale examined PA levels across three dimensions: intensity, duration of a training session, and frequency. The score for the amount of PA = intensity × (score for the duration of each workout–1) × frequency of workouts, where each aspect is divided into five levels (counting 1–5 points), and the amount of PA is based on one scale rated from to 0–100. Based on this information, the activity level score was calculated, with higher scores indicating higher activity levels. The scale used in this study has been widely used in PA research and has good validity and reliability. Cronbach’s *α* coefficient of the scale was 0.84, which indicates good reliability (30).

#### 2.3.8 Physical fitness measurement

Physical fitness was measured in accordance with the National student physical health standard for College Students using the following items and values (31): Body Mass Index (BMI): 15%; BMI = weight (kg)/height^2^ (m^2^). Vital capacity: 15%; 50 meter dash: 20%; Sit and reach: 10%; Standing long jump: 10%; Pull-ups (male)/1 min or sit-ups (female): 10%; 1,000 meter run (men)/800 meter run (women): 20%. Evaluation Criteria: Excellent: Overall score of 90.0 and above. Good: 80.0–89.9 points. Passed: Total score 60.0 ~ 79.9. Failed: Total score of 59.9 or less. These items fully reflect students’ physical condition, physical function, and physical quality.

#### 2.3.9 Psychological well-being measurement

The Chinese version of the Psychological Well-Being Scale (PWBS) revised by Xing and Huang (2004) was used to measure the psychological well-being of college students (32). The scale consists of 18 items, including six dimensions: independence, environmental control, good relationships, personal growth, life goals, and self-acceptance. It is based on a 6-point Likert scale, where 1–6 means “not consistent at all,” fairly consistent, somewhat inconsistent, somewhat consistent, fairly consistent, and completely consistent, respectively. There were no reverse-scored questions on the scale. The higher the total score, the higher the individual’s psychological well-being. The Cronbach’s alpha coefficient for the scale in this study was 0.88 (32).

#### 2.3.10 Psychological resilience measurement

This study used the Chinese version of the method described by Yu et al. Revised Psychological Resilience Scale. (33), which consists of 25 items with three dimensions: strength, resilience, and optimism. A 5-point Likert scale was used, ranging from 0 (not at all) to 4 (almost always), with higher total scores representing higher levels of individual psychological resilience. In this study, Cronbach’s alpha coefficient for the scale was 0.96 (33).

#### 2.3.11 Screen time measurement

There are a variety of methods for measuring screen time, including self-reporting, device monitoring, and observation. Self-report is a method in which participants record the time they spend using electronic devices and the content of their activities on a daily or weekly basis. Device monitoring is a method in which time and behavior are automatically recorded through software or applications installed on electronic devices, and observation is a method of estimating screen time by directly observing the behavior of people using the devices. This study used a self-report method in which participants reported “how much time they spent in front of electronic screens each day,” which was categorized into nine levels ranging from “none,” to about 15 min or less, to about 30 min, about 1 h, about 2 h, about 3 h, about 4 h, and about 5 h to about 6 h or more, where the unit of calculation is the hour.

### 2.4 Data acquisition and quality control

In the 2022–2023 school year, students received a questionnaire and a physical fitness test at the beginning of the school year (September). The questionnaire was administered through a paper version of the test booklet, and the physical fitness test was conducted in the school gymnasium by a professional using a standardized procedure. Before data collection, the researcher explained in detail the purpose, procedure, and precautions of the study to the students to ensure their informed consent. All data were collected and recorded by trained researchers to ensure accuracy and reliability. The questionnaire administration process took approximately 30 min, and all questionnaires were collected on-site.

To ensure the quality of the study, the following measures were taken: Sampling was carried out strictly according to the sampling plan to ensure the representativeness and randomness of the samples. Researchers were trained to become familiar with the purpose, methodology, and process of the study, and to master data collection and measurement techniques. Standard operating procedures were strictly followed during data collection to ensure data accuracy and reliability. Data were double-entered and verified to minimize data entry errors. Before the data analysis, the data were cleaned and checked to remove abnormal and missing data. The research results were reviewed and discussed multiple times to ensure their reliability and validity. The above measures made it possible to effectively control the quality and improve the credibility and validity of the study.

### 2.5 Data analysis

Organizing data from questionnaires, field tests, or other data collection methods. Perform data cleansing, including handling missing values, outliers, and duplicate records. Statistical analysis of the data was performed using SPSS software (version 26.0). Descriptive statistical analysis was applied to the initial examination of the data set, including calculating the mean, standard deviation, skewness, and kurtosis to understand the basic characteristics and distribution of the data. Frequency distribution tables and histograms were created to understand the distribution properties of the data. Check the assumptions regarding normality, linearity, homoscedasticity, and independence of the data. Visualization tools, such as box-and-line plots and scatter plots, are applied to identify patterns and trends in the data. Analyze the reliability of the questionnaire data and evaluate the internal consistency of the scale. B. Cronbach’s alpha coefficient. The linear correlation between variables was analyzed using the Pearson’s correlation coefficient. The strength and direction of the association between the variables were evaluated. A t-test and analysis of variance (ANOVA) were used to analyze the differences in PL between different groups and identify the effects of factors such as sex, age, and place of origin on PL.

## 3 Result

### 3.1 KMO test and correlation analysis

The results of the KMO test are presented in Table 1. The KMO sampling adequacy measure was 0.866, indicating that the data were well-suited for factor analysis. This means that there is a strong correlation between the variables and the data structure can be simplified by extracting the principal components to reveal the underlying patterns of the data. The approximate chi-square value of Bartlett’s sphericity test is 1938.814, with 28 degrees of freedom and a significance of 0.000, further confirming that the data are suitable for factor analysis.

**Table 1:** Results of the KMO test.

KMO and Bartlett’s test		
KMO Quantity of Sample Suitability		0.859
Bartlett’s test of sphericity	Approximate chi-square value	1910.334
	Degrees	28
	Significance	0.000

The correlation coefficient matrix of the dataset was calculated to determine the correlation between the data features. The results are presented in Table 2. The correlation coefficient matrix shows the correlation between physical fitness, PA, exercise attitude, exercise motivation, exercise confidence, body appreciation, exercise willpower, and exercise commitment. By analyzing the correlation coefficient matrix, the strength and direction of the relationship between the variables can be understood. For example, the correlation coefficient between physical fitness and PA is 0.103, indicating that there is a positive correlation between the two, and the correlation coefficient between exercise attitude and commitment is 0.740, indicating that these two variables have a strong positive correlation. This correlation information provides an important reference base for constructing the PL index and helps to determine which variables have a greater influence on PL and how these variables can be combined to assess the level of PL.

**Table 2:** Matrix of correlation coefficients between variables.

Variables	Physical fitness	PA	Exercise attitude	Exercise motivation	Exercise confidence	Body appreciation	Exercise willpower	Exercise commitment
Physical Fitness	1	0.103^**^	0.167^**^	0.103^**^	0.154^**^	0.183^**^	0.122^**^	0.158^**^
PA		1	0.546^**^	0.367^**^	0.416^**^	0.204^**^	0.237^**^	0.568^**^
Exercise Attitude			1	0.542^**^	0.542^**^	0.456^**^	0.458^**^	0.740^**^
Exercise Motivation				1	0.331^**^	0.289^**^	0.259^**^	0.470^**^
Exercise Confidence					1	0.252^**^	0.357^**^	0.587^**^
Body Appreciation						1	0.416^**^	0.401^**^
Exercise Willpower							1	0.499^**^
Exercise Commitment								1

*p < 0.05; **p < 0.01.

The correlation between the data features was determined by calculating the covariance matrix or correlation coefficient matrix of the dataset. The results of the KMO test showed that the data were suitable for factor analysis, indicating a certain degree of correlation between the variables, which provided a basis for the extraction of principal components.

### 3.2 Calculation of eigenvalues and eigenvectors

The eigenvalues and corresponding eigenvectors of the covariance matrix were calculated. The eigenvalues represent the amount of variance explained by each principal component, and the eigenvectors represent the direction of the principal components. The results of the eigenvalues and percentage of variance explained are presented in Table 3, where the eigenvalues indicate the amount of variance explained by each principal component. In this study, the eigenvalues of the first four principal components were 3.678, 1.007, 0.887, and 0.722, respectively, which corresponded to 45.976, 12.593, 11.093, and 9.026% of the explained variance, and the cumulative percentage of variance explained was 78.688%. Typically, the decision on the number of principal components to select can be based on the cumulative contribution (for example, 70–80%). Principal components can also be selected based on an eigenvalue greater than 0.7 (34). In this study, the percentage of cumulative variance explained by the first four principal components was close to 80%; therefore, the first four principal components were selected for the analysis. These four main components reduced the dimensionality of the data while retaining most of the data variability, making the analysis more concise and effective.

**Table 3:** Percentage of eigenvalues and variance explained in the principal component analysis of PL.

Total variance explained						
Component	Initial eigenvalues			Extracted loadings sum of squares		
	Total	Variance percentage	Cumulative %	Total	Variance percentage	Cumulative %
1	3.678	45.976	45.976	3.678	45.976	45.976
2	1.007	12.593	58.569	1.007	12.593	58.569
3	0.887	11.093	69.662	0.887	11.093	69.662
4	0.722	9.026	78.688	0.722	9.026	78.688
5	0.578	7.230	85.918			
6	0.526	6.576	92.494			
7	0.354	4.423	96.917			
8	0.247	3.083	100.000			

Extraction method: Principal Component Analysis.

The explained eigenvalues and variance percentage not only determine the number of principal components but also provide important clues for understanding the structure of the PL. Each principal component represents a potential dimension of the data, and by analyzing the eigenvalues and percentage of variance explained by the principal components, it is possible to understand the extent to which each dimension contributes to the PL. For example, the percentage of variance explained for the first principal component was 45.976%, indicating that this principal component has a strong ability to explain PL and may contain important factors that are closely related to PL. By further analyzing the stresses on the principal components, it is possible to accurately determine these important factors, thereby obtaining a deeper understanding and basis for constructing the PL index.

### 3.3 Select the principal component

The top principal components that explained most of the variance were selected based on the magnitude of the eigenvalues. The number of main components to be chosen can usually be determined by the cumulative contribution rate (for example 70–80%). The results are presented in Table 4.

**Table 4:** Component matrix after principal component analysis of PL after standardization.

Component matrix				
Variables	Component			
	1	2	3	4
Exercise Attitude	0.872	−0.085	−0.001	0.076
Exercise Commitment	0.870	−0.114	0.011	−0.119
Exercise Confidence	0.704	−0.131	0.155	−0.405
PA	0.670	−0.354	0.311	−0.053
Exercise Motivation	0.642	−0.178	0.074	0.624
Exercise Willpower	0.629	0.261	−0.461	−0.294
Body Appreciation	0.581	0.442	−0.422	0.243
Physical Fitness	0.265	0.741	0.609	0.002

Extraction Method: Principal Component Analysis. a. Four components were extracted from the extract.

### 3.4 Principal component analysis

The results of the rotated component matrix of the principal component analysis are shown in Table 5. The rotated component matrix of principal component analysis shows the loadings of each variable on the four principal components. These loadings reflect the strength and direction of the relationship between the variables and principal components. By analyzing the loadings, the role of each variable in PL as well as the actual importance of each principal component can be better understood. The rotated component matrix of principal component analysis not only helps to understand the relationship between the variables and the principal components, but also provides a detailed understanding of the structure of PL. By analyzing the loadings of each variable on different principal components, it is possible to determine how different aspects of PL are distributed in the principal components. As shown in Table 5, the eight factors were analyzed by principal component analysis to form four principal components, of which the first principal component consisted of four factors: exercise confidence, PA, exercise engagement and exercise attitude, and the four theoretical dimensions of “cognition, emotion, will and action,” which are closer to “emotion” and expressed as emotions and affections of participation in PA. The second main component is body appreciation and exercise willpower, which reflects the “will” in the four dimensions of cognition, emotion, will, and action, and indicates the willpower and willingness to participate in PA, which is more consistent with the theoretical dimensions. The third component is exercise motivation, which is divided into four dimensions: cognition, emotion, will, and action. Cognition and intention to participate in PA, which is close to “cognition.” The fourth main component consists of physical fitness, which is closer to “action” in the four dimensions of cognition, emotion, will, and action, that is, h the behavioral action of PA. When comparing the theory-driven model of cognition, emotion, will, and action with the data-driven model, the objective data model corrected the theory-driven model; therefore, we used the results of principal component analysis to construct the PL index.

**Table 5:** Component matrix after principal component analysis rotation.

Variables	Component			
	Factor 1	Factor 2	Factor 3	Factor 4
Exercise Confidence	0.807			
PA	0.746			
Exercise Commitment	0.744			
Exercise Attitude	0.640			
Body Appreciation		0.792		
Exercise Willpower		0.789		
Exercise Motivation			0.865	
Physical Fitness				0.987

### 3.5 Constructing weighting models

The original data were projected onto these principal components to obtain a new dimensionally reduced dataset. The weight of each principal component can be determined from its intrinsic value. Normally, the larger the eigenvalue, the higher the weight of the corresponding principal component. The construction of the centimeter-weighting model takes place in five steps.

Step 1: Determine the coefficients of the principal components for each linear combination. Coefficient = number of loadings/open squares of the corresponding eigenvalues. Coefficient of exercise attitude = 0.872/ (3.678)1/2 ≈ 0.455 (Table 6).

**Table 6:** Coefficients in the linear combination after principal component analysis.

Variables	Factor 1	Factor 2	Factor 3	Factor 4
Exercise Attitude	0.455	−0.085	−0.001	0.090
Exercise Commitment	0.454	−0.114	0.011	−0.140
Exercise Confidence	0.367	−0.131	0.165	−0.476
PA	0.349	−0.353	0.330	−0.062
Exercise Motivation	0.335	−0.178	0.078	0.735
Exercise Willpower	0.328	0.260	−0.489	−0.346
Body Appreciation	0.303	0.440	−0.448	0.286
Physical Fitness	0.138	0.739	0.646	0.003

Step 2: The coefficients of each factor in the composite scoring model are determined. Composite coefficient = {factor (component 1) *corresponding percentage of variance (component 1) + factor (component 2) × corresponding percentage of variance (component 2) + + factor (Component N) *corresponding percentage of variance (component N)}/Cumulative Percentage of Variance For example, the composite coefficient of factor 1 = {0.455*45.976% + (−0.085) *12.593% + (−0.001) *11.093% + (0.090) *9.026%}/ 78.688% ≈ 0.262. The results are presented in Table 7.

**Table 7:** Coefficients of the variables in the composite score model.

Variables	Composite coefficient
Exercise Attitude	0.262
Exercise Commitment	0.232
Exercise Confidence	0.162
PA	0.187
Exercise Motivation	0.306
Exercise Willpower	0.124
Body Appreciation	0.217
Physical Fitness	0.290

Step 3: Determine the factor weights Normalize the coefficients of each factor in the composite score model, weight = factor composite coefficient/sum of the composite coefficients of each factor. For example, the weight of factor1 = 0.262/ (0.262 + 0.232 + 0.162 + 0.187 + 0.306 + 0.124 + 0.217 + 0.290) ≈ 0.147. The details are listed in Table 8.

**Table 8:** Weighting coefficients for each factor.

Variables	Composite coefficient	Weighting coefficients
Exercise Attitude	0.262	0.147
Exercise Commitment	0.232	0.130
Exercise Confidence	0.162	0.091
PA	0.187	0.105
Exercise Motivation	0.306	0.171
Exercise Willpower	0.124	0.070
Body Appreciation	0.217	0.122
Physical Fitness	0.290	0.163

### 3.6 Assignment of weights

Weighting coefficients reflect the extent to which each factor contributes to the PL index. In this study, as shown in Table 8, Exercise Attitude, Exercise Commitment, Exercise Confidence, PA, Exercise Motivation, Exercise Willpower, Body Appreciation, and Physical Fitness The weights of the factors are 0.147, 0.130, 0.091, 0.105, 0.171, 0.070, 0.122, and 0.163, respectively, and the determination of these weight coefficients provides an important basis for the construction of the PL Evaluation Index Model, which can more accurately reflect the actual level of PL of college students. At the same time, the weighting coefficients can also help improve college students’ PL. The variance contribution of each principal component is used as the weight in the model. A test dataset was used to validate the model and verify that the model accurately reflected the characteristics of the original data.

Step 4: Determine the PL evaluation model. PL Index Evaluation Model = 0.147*Exercise Attitude Standard Score+0.130*Exercise Commitment Standard Score+0.091*Exercise Confidence Standard Score+0.105*PA Standard Score+0.171*Exercise Motivation criterion score+0.070* Exercise Willpower criterion score+0.122*Body Appreciation criterion score+0.163* Physical Fitness criterion score.

Step 5: Standardize the PL index model for Chinese college students. Standardized percentile X_norm_ = (X − Xmin)/(Xmax−Xmin) × 100. where X_norm_ is the value after standardization, X the original data, X_min_ the minimum value of the standardized data, and X_max_ the maximum value of the standardized data. In this study, X_max_ = 1.8891, X_min_ = −2.2971. After standardizing the percentages, 1.8891 and-2.2971 correspond to a percentage of 100 and 0 points, respectively. The PL index model for Chinese college students was standardized to make the results more intuitive and easier to compare. Standardized results can better reflect the relative level of college students’ PL and provide a convenient way to assess and compare the PL of different college students.

### 3.7 Calibration validity of PL evaluation models

The PL evaluation index model was constructed based on the results of the principal component analysis. The weight coefficients of each factor, exercise attitude, commitment, confidence, PA, motivation, willingness, appreciation, and fitness were determined. These factors were combined to construct the PL assessment index model. This model was constructed based on the structure of PL and the relationship between the factors, as revealed by principal component analysis, as well as the degree of contribution of each factor to PL. Therefore, the model has a certain rationality and science. We tested the validity of the calibration association of the college students’ PL index by calculating the correlations between the index and other variables (screen time, mental resilience, and psychological well-being). The results show that college students’ PL index is significantly negatively correlated with screen time, with a correlation coefficient of −0.257, while the college students’ PL index is positively correlated with the other variables, with correlation coefficients reaching a significant level (*p* < 0.05) and a minimum correlation coefficient of 0.594, indicating that the college students’ PL index has a better correlation effectiveness with the calibration scale.

The PL evaluation index model can be a scientific and effective tool for assessing college students’ PL. By converting college students’ performance on each factor into standardized scores and weighting them and summing them according to the weighting coefficients, a comprehensive PL evaluation index can be obtained. This index can intuitively reflect the level of college students’ PL and provide an important reference for the reform of physical education in colleges and universities, the formulation of health promotion policies, and individual self-assessment. At the same time, the standardized processing of the model makes the results more intuitive and easier to compare, allowing horizontal comparison between different college students and vertical comparison of the PL of the same college students at different points in time to better understand the trend of change and development of PL.

### 3.8 Assessment of PL index of Chinese college students

#### 3.8.1 Assessment and analysis of PL of Chinese college students

**Figure 1:**
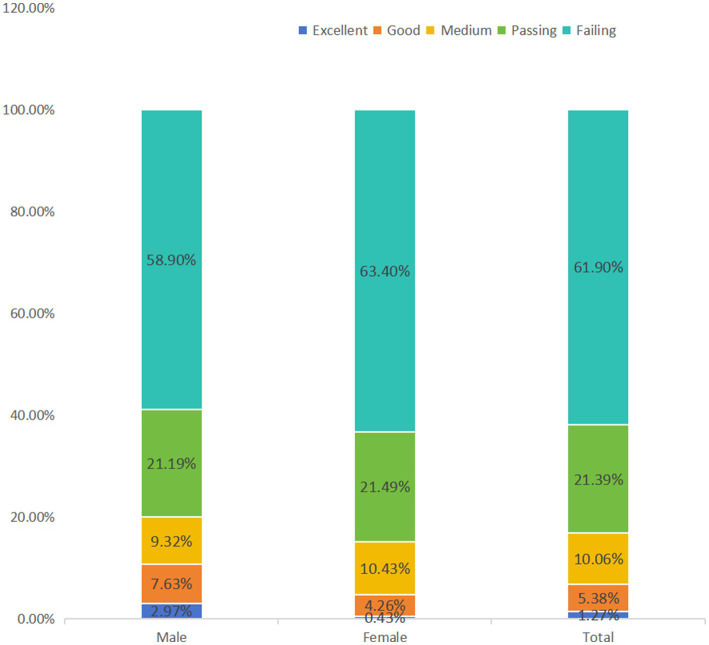
Gender differences in PL competency assessments of Chinese college students.

Chinese college students’ PL scores were assessed on a five-level scale categorized as excellent (≥ 90), good (80–89), moderate (70–79), passing (60–69), and failing (below 60). The results are shown in Table 9, which shows that the overall level of PL among Chinese college students was not high, as shown in the table of the five-level assessment of PL. The proportions of excellent and good grades were low at 1.27 and 5.38%, respectively; the proportions of moderate, passing, and failing grades were 10.06, 21.39, and 61.90%, respectively. This indicates that the PL of most college students is below the medium level, more than 60% are at the failing level, and the PL of college students needs to be further improved. In addition, as shown in Figure 1, the gender difference is not significant: from the perspective of gender, the distribution of male and female college students in the five-level assessment of PL is relatively similar. Among the male college students, the proportions of excellent, good, moderate, passing, and failing were 3.39, 7.63, 10.59, 20.76, and 57.63%, respectively. Among female college students, the corresponding proportions were 0.64, 2.55, 12.55, 17.45, and 66.81%. Although the proportion of male college students with excellent and good grades was slightly higher than that of female college students, the difference was not statistically significant.

**Table 9:** Correlation matrix between variables of calibration validity.

Variables	Screen time	Psychological resilience	Psychological well-being	PL index
Screen time	1			
Psychological resilience	−0.146^**^	1		
Psychological well-being	−0.090^*^	0.742^**^	1	
PL Index	−0.257^**^	0.595^**^	0.594^**^	1

*p < 0.05; **p < 0.01.

#### 3.8.2 Assessment and analysis of physical fitness and health tests for college students in China

**Figure 2:**
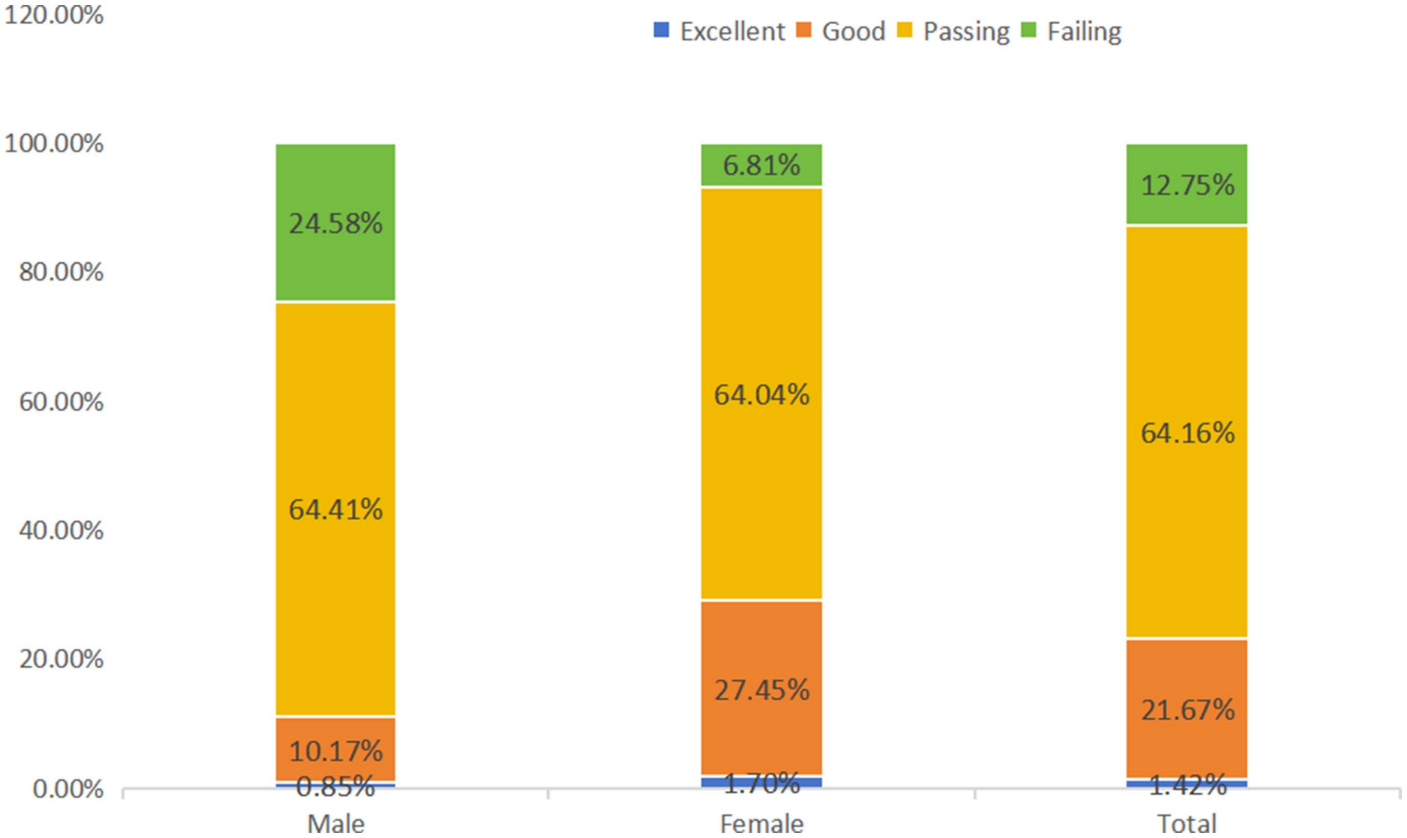
Assessment of physical fitness and health test level 4 for Chinese college students.

The results, as shown in Figure 2, have a high passing rate, but a low proportion of excellent and good grades. The table of the four-level assessment of the physical fitness test of Chinese college students shows that the passing rate of the physical fitness test of college students was high, at 64.16%, but the proportion of excellent and good grades was relatively low, at 1.42 and 21.67%, respectively. This indicates that the physical fitness of college students is generally at an intermediate level, but there is still room for improvement. A comparison with the five-level assessment of PL shows that the physical fitness test is an important aspect of PL assessment, which mainly reflects the performance of college students in terms of their physical functions and athletic abilities. Compared with the five-level assessment of PL, the physical fitness test pays more attention to the measurement of objective indicators such as BMI, vital capacity, 50-meter run, and 1,000-meter run. However, PL includes not only physical fitness but also psychological factors such as Exercise Motivation, Exercise Commitment, and Exercise Confidence, as well as will factors such as body appreciation and exercise willpower. Therefore, the results of the physical fitness test and the five-level assessment of PL are both related and different. Students with better physical fitness test results may also have higher scores on the PL assessment, but students with high PL may not necessarily perform well on the physical fitness test because PL also includes psychological and will factors. The results of physical fitness tests provide an important reference for physical education in colleges and universities and the health management of college students. According to the results of the physical fitness test, colleges and universities can adjust the physical education curriculum and teaching content and strengthen the monitoring and management of students’ physical fitness. Simultaneously, college students can formulate personalized exercise plans based on physical fitness test results to improve their overall physical fitness level.

### 3.9 Comparative analysis of PL indices of college students of different genders

The results of the analysis of the PL indices of college students of different genders showed that the mean values of the PL indices of male college students were slightly higher than those of female college students, which were 56.0508 ± 18.4067 and 53.8511 ± 14.5370, respectively. However, the test of variance showed that the PL indices of college students of different genders were not significantly different from one another (*t* = 1.730, *p* > 0.05). This suggests that gender has less influence on college students’ PL, but male college students may have a slight advantage in PL.

### 3.10 Differences and analysis of PL index of college students of different ages

The results, as shown in Table 10, indicate that there are some differences in the PL index of college students of different ages, but the overall fluctuation is not significant. In terms of specific data, the mean value of the PL index of 17-year-old college students was the lowest at 45.000 ± 14.6697, and the mean value of the PL index of 24-year-old college students was the highest at 59.2857 ± 25.0447. However, the differences in the PL index of college students of different ages were not significant by one-way ANOVA (*F* = 1.040, *p* > 0.05). Differences in the PL indices of college students of different ages may be related to factors such as college students’ growing environment, study pressure, and lifestyle.

**Table 10:** PL index of college students of different ages.

Age	n	M	SD	SE	95% CI	
					Lower limit	Upper limit
17.00	6	45.0000	14.66970	5.98888	29.6051	60.3949
18.00	106	57.1792	14.91104	1.44829	54.3076	60.0509
19.00	157	53.7643	15.53620	1.23992	51.3151	56.2135
20.00	105	52.8000	14.16754	1.38261	50.0582	55.5418
21.00	195	55.0667	16.63435	1.19121	52.7173	57.4161
22.00	105	53.7619	16.56138	1.61623	50.5569	56.9669
23.00	20	56.9000	17.05996	3.81472	48.9157	64.8843
24.00	7	59.2857	25.04472	9.46602	36.1232	82.4482
25.00	5	57.2000	27.04071	12.09297	23.6245	90.7755
总计	706	54.5864	15.95611	0.60052	53.4074	55.7654

### 3.11 Assessment and comparison of PL index of college students from different birthplaces

The results showed that the mean values of the PL indices of college students from rural origins were slightly higher than those of college students from urban origins (55.0316 ± 14.4509 and 54.3377 ± 16.1542, respectively). However, the test of variance showed that the differences in PL indices of college students from different origins were not significant (*t* = −0.554, *p* > 0.05). The insignificant difference in the PL index between college students from rural and urban birthplaces may be related to the development of modern society and equalization of educational resources. Although the differences in PL indices among college students from different places of origin are not significant, the influence of the place of origin factor can still be considered in physical education and health promotion. For example, diversified physical activities and health education can be carried out to address the different characteristics of rural and urban students to increase students’ participation and motivation. Simultaneously, sports exchanges and cooperation between urban and rural areas can be strengthened to promote the sharing of educational resources and balanced development.

## 4 Discussion

This study analyzed in depth the construction and assessment of the PL index for Chinese college students. The PL index constructed through principal component analysis and other methods has a certain degree of scientific validity and can reflect the actual level of college students’ PL more accurately. However, the assessment results also show that the overall level of college students’ PL is not high, and the differences in PL among college students of different genders, ages, and places of origin are insignificant. This suggests that college universities and society should pay more attention to physical education and health promotion for college students and take targeted measures to improve their PL. Meanwhile, future studies can further expand the sample range and improve measurement tools and research methods to gain a more comprehensive and in-depth understanding of the status of college students’ PL and its influencing factors.

### 4.1 Assessment of PL index of Chinese college students

China currently lacks assessment tools for college students’ PL, and even the existing assessment tools are limited to self-reported scales, which in essence test perceived PL (10, 35). There is no evaluation standard or index scale for college students’ PL. Colleges and universities can use the PL Index to develop health promotion strategies and curricula. The college students’ PL index constructed in this study uses an all-round test, both psychological scale measurements and field measurements in the form of college students’ physical fitness test content as the main form, which is more reflective of college students’ physical fitness and athletic ability. The college students’ physical fitness test is very holistic, and it is a multidimensional index to comprehensively measure college students athletic ability. In this study, it is appropriate to use the word “action” to represent the behavior and actions of college students. At present, scholars in China have developed a PL questionnaire for college students, which is initially divided into four domains: emotional, physical, cognitive, and behavioral. The final questionnaire consists of three domains (physical and behavioral domains, cognitive domain, and emotional domain) and seven dimensions (fundamental movement skills, motor skills, physical activity, cognition of PA, cognition of healthy lifestyles, motivation to participate in physical activity, and confidence to participate in physical activity) comprising a total of 38 items (35). However, its PL Scale for College Students tests not college students’ PL but the Perceived PL Questionnaire. This study’s physical abilities are more objective and realistic as the results are derived from specific sports and field tests, and the domains and dimensions are identified and weighted through principal component analysis, which, although not a perfect fit with the indicators constructed by the theory of “cognition, emotion, Will, and action,” most of the overlap suggests that data-driven assessment guides theory-driven assessment, and this study ultimately follows theory-driven assessment (35). The assessment and results of the data-driven index were used in this study. The validity and reliability of the PL index were demonstrated using a calibration correlation validity test. After the college students’ PL index was converted to standardized percentages, its evaluation criteria showed greater consistency with the college students’ physical fitness test, which fully demonstrated the reliability and validity of the index. The results of the evaluation indicate that the PL level of college students is worrying, and education departments and policymakers must take measures to improve the PL level of college students.

### 4.2 The importance of constructing the PL index

This study is the first index assessment of PL among Chinese college students, which not only helps to promote the precision of physical education reform and the rational allocation of university resources, but also can provide an objective basis for college students’ self-perceptions. The findings support the development of college students’ PL as part of a holistic approach to supporting their well-being and mental health, and provide new perspectives on interventions to improve their mental health, and physical education programs can play an important role in this process by designing courses that focus on the concept of PL to improve their physical and mental health (36). The construction of the PL index provides specific directions and goals for physical education reform. The education department can adjust the content of physical education courses and teaching methods in a targeted manner according to the weights of the factors in the index. Universities could integrate the PL Index into health screenings, and ministries might adopt it for national student health surveillance (37). For example, if college students are found to score low in Exercise Motivation, activities such as inspirational stories of sports stars and fun sports competitions can be added to the physical education curriculum to stimulate students’ interest in and motivation to exercise. Meanwhile, for factors with higher weights, such as Physical Fitness and Exercise Attitude, teaching resources can be increased to improve the teaching quality. Colleges and universities can reasonably allocate resources such as sports facilities and teachers based on the assessment results of the PL index. More personalized guidance and support can be provided for student groups with lower levels of PL, such as by opening special fitness counseling courses and organizing small sports clubs. This not only improves the efficiency of resource utilization, but also better meets the diverse needs of students and enhances the overall PL level. The PL index allows college students to understand their PL status objectively. Students can clarify their strengths and weaknesses in various aspects through an index to formulate personalized exercise plans and development goals. For example, if they find that they score high in body appreciation but lack exercise perseverance, they can focus on cultivating their perseverance, such as setting specific exercise goals and finding exercise partners to supervise each other to gradually improve their PL.

### 4.3 Impact of the PL index assessment on the health of college students

From the results, it can be seen that the overall situation of Chinese college students’ PL index scores is not optimistic, showing a consistent development trend with annual college students’ Physical Fitness results (7). The college students’ PL Index standard not only enables them to enhance their ability to cope with stress and cultivate healthy habits, but also drastically reduces future health risks. Good PL helps college students cope better with stress in their studies and lives. Physical exercise can promote the secretion of endorphins and other neurotransmitters, which can improve the emotional state and mental toughness. One study found that interventions targeting PL exercises may be effective in improving college students’ sense of well-being and in reducing psychological distress. Additionally, this research suggests that integrating PL components into physical education and activity programs may help meet an individual’s overall health needs (38). When college students face challenges such as academic stress and relationship problems, those with good PL can cope more comfortably and reduce negative emotions, such as anxiety and depression. For example, during exam weeks, students who regularly participate in PA may show better mental states and coping abilities. The improvement in PL is not only reflected in physical exercise but also leads college students to develop healthy living habits. Physically active students tended to pay more attention to a balanced diet and routine. They are more likely to choose healthy foods such as fruits, vegetables, and whole grains, and avoid excessive intake of high-calorie, high-fat, and high-sugar foods. At the same time, they also pay more attention to getting enough sleep to ensure that their bodies get sufficient rest and recovery. These healthy living habits have a positive impact on the long-term health of college students. Developing good PL during college can lay a solid foundation for future health. Studies have shown that a healthy lifestyle and good PL at a young age can reduce the risk of developing chronic diseases such as cardiovascular disease, diabetes, and obesity in adulthood (19). By improving PL, college students can enhance their immunity and metabolic functions and reduce the damage caused by poor lifestyles, thus reducing future health risks.

### 4.4 Implications of the PL index assessment for education

The reality of the overall low PL index among Chinese college students provides a policy rationale for educational administrations to not only incorporate the concept of whole-person education, but also to strengthen the relevance of faculty building as well as to create a positive campus sports culture. PL cultivation should be integrated into the concept of whole-person education in colleges and universities. Whole-person education emphasizes the comprehensive development of students, including intellectual, emotional, physical, social, and other aspects. Incorporating PL into the education goal system can enable physical education and education in other disciplines to integrate and jointly promote the overall growth of students. For example, in ideological and political education courses, sportsmanship and values can be combined to cultivate students’ qualities, such as perseverance and teamwork; in the teaching of professional courses, elements of sports activities, such as group competitions and outdoor practice, can be appropriately introduced to enhance students’ interest and participation in learning. To better cultivate the PL of college students, colleges and universities need to strengthen the construction of physical education teacher teams. According to the requirements of the PL Index, physical education teachers are provided with professional training to improve their abilities in Exercise Motivation stimulation, psychological counseling, and teaching of sports skills. At the same time, physical education teachers are encouraged to cooperate with teachers of other disciplines to carry out interdisciplinary teaching and research activities to provide students with more comprehensive educational services. For example, physical education teachers can be organized to cooperate with psychology teachers to carry out sports psychological counseling courses to help students overcome psychological barriers in exercise and improve the effect of exercise. Campus sports culture plays an important role in promoting the cultivation of PL among college students. Colleges and universities can create a positive campus sports culture atmosphere by organizing various sports events, activities, and lectures. For example, organizing campus marathons, basketball leagues, fitness lectures, and other activities can attract more students to participate in sports activities. Simultaneously, students are encouraged to set up sports societies and clubs to carry out independent sports activities and cultivate their sports interests and hobbies. In addition, campus media can be used to publicize sports and health knowledge and the deeds of outstanding sports figures to stimulate student enthusiasm and motivation for exercise. The close relationship between PL and PA indicates that the PL Index can act as a core indicator for detecting and measuring the level of PA of college students, and the PL Index of college students can be used as an important criterion for the effectiveness of exercise intervention. It has been shown that by learning novel motor skills in a fun and engaging group setting, participants in the control condition completed only baseline and follow-up assessments (39). In contrast, all participants completed the baseline and follow-up assessments of motor competence, motivation, knowledge, and confidence (40). The results revealed significant condition interaction times for PL, suggesting that an environmental context-based exercise intervention can be effective in improving overall PL among college students.

### 4.5 Value of the PL index assessment to society

Improving college students’ PL is one way to maintain an active lifestyle (39). Understanding the specifics of college students’ PL not only contributes to the development of socially responsible citizens and the promotion of the sports industry but also promotes social harmony and stability and improves the quality of life of college students. It even affects their quality of life in adulthood and old age (41). The importance of PL in buffering college students from declining quality of life has been emphasized by the findings that physical ability, motivation for PA, and environmental interactions are strongly associated with higher quality of life, and that cultivating PL encourages lifelong responsible participation in PA, which promotes quality of life even during COVID-19 pandemic restrictions (42). By fostering students with higher levels of PL, that is, the ability to take more self-responsibility for participating in lifelong PA by recognizing their well-rounded and unique experiences as well as creating a more empowering and collaborative athletic environment, college students not only develop a sense of social responsibility and teamwork through participation in PA but also enhance their quality of life (20). Team programs in sports activities require students to learn to cooperate with others, respect others, and follow rules that are crucial for them to become socially responsible citizens. For example, in volunteer service activities, college students with good PL and teamwork can play their roles better and contribute to society. The improvement in college students’ PL will drive the growth of sports consumption and promote the development of the sports industry. As college students pay more attention to physical exercise, their demand for sports goods, fitness services, sports events, and so on will continue to increase. This will provide a broad market space for the sports industry and promote its innovation and development of the sports industry. Meanwhile, the development of the sports industry will also provide more sports resources and employment opportunities for college students, thereby forming a virtuous circle. Healthy college student groups are an important foundation for social harmony and stability. A good PL can improve the physical and mental health of college students and reduce the occurrence of social contradictions and conflicts. At the same time, college students who actively participate in sports activities can better integrate into society, enhance social interaction and communication ability, and promote exchange and cooperation between different groups. For example, in community sports activities, college students can participate in sports exercises with residents, enhance neighborly relations, and promote harmonious development of the community.

### 4.6 Limitations

This study has several limitations. First, the sample was restricted to undergraduate students in Shanghai, an international metropolis with distinct socioeconomic, cultural, and sports resource characteristics, limiting the generalizability of findings to college students in other regions or educational levels, who may differ in academic pressures, lifestyles, and PL influences (42). Second, reliance on self-reported questionnaires and scales introduced potential biases, such as memory inaccuracies and social desirability effects, while cultural, age, and gender factors might compromise measurement reliability. The PL assessment tools, though multidimensional, may incompletely capture aspects like sports safety awareness or cultural literacy. Methodologically, the quantitative approach lacked depth in exploring subjective experiences toward physical activity, necessitating complementary qualitative methods. Additionally, the cross-sectional design only provided a snapshot of PL status, failing to track its dynamic, long-term development influenced by multifactorial processes.

### 4.7 Directions for future research

Future research should expand geographically and demographically to improve understanding of physical literacy (PL). Cross-regional and cross-country comparisons can reveal how cultural, educational, and resource differences affect the learning performance of college students, thus contributing to the development of global strategies. Research should be extended to other age groups (e.g., adolescents, older adults) to identify life-stage-specific PL needs and inform lifelong interventions. In addition, focusing on marginalized student populations (e.g., minorities, students with disabilities) is critical to addressing unique challenges and designing tailored support programs. Integrating these multidimensional approaches-across different regions, age groups, and disadvantaged populations-will enable holistic, equity-driven PL strategies and evidence-based policy development. Future research is also needed to test whether increased PL has a positive impact on PA levels. In future research, we will integrate measurement data from objective devices such as wearable devices and accelerometers, or data from observer ratings, in order to reduce the possible influence of social expectation bias, recall bias and cultural factors.

## 5 Conclusion

This study constructed a Physical Literacy (PL) index for Chinese college students via principal component analysis, integrating dimensions like Exercise Attitude, Commitment, Confidence, Physical Activity, Motivation, Perseverance, Physical Appreciation, and Fitness. Validated by statistical methods (KMO test, variance explanation, rotated component matrix), the model demonstrated scientific rigor. Assessments revealed a generally low PL level, with high failure rates and minimal excellence across five-tiered evaluations, though differences by gender, age, or origin were negligible. While physical fitness had high pass rates (four-tiered assessment), excellence remained limited, highlighting a baseline of physical health but substantial potential for PL improvement.

The content and structure of the PL Assessment Tool (PLAT) for Chinese college students, which was constructed based on the traditional educational idea of “knowledge, emotion, will and behavior,” is scientific and valid. The instrument comprehensively assesses the PL of college students in four dimensions: cognition, emotion, will, and behavior, and provides an effective method and way to deeply understand the PL of college students. Through the measurement of different dimensions, it can provide targeted suggestions for the improvement of college students’ PL, and promote college students to develop a healthy lifestyle and positive PA habits. At the same time, this test method also provides a new assessment tool for schools, communities fitness organizations, etc., which helps to promote the improvement of PL for all people.

## Data Availability

The raw data supporting the conclusions of this article will be made available by the authors, without undue reservation.

## References

[1] Corbin CB. Implications of physical literacy for research and practice: a commentary. Res Q Exerc Sport. (2016) 87:14–27. doi: 10.1080/02701367.2016.1124722, PMID: 26889581

[2] Tremblay MS Colley RC Saunders TJ Healy GN Owen N. Physiological and health implications of a sedentary lifestyle. Appl Physiol Nutr Metab. (2010) 35:725–40. doi: 10.1139/H10-079, PMID: 21164543

[3] Edwards LC Bryant AS Keegan RJ Morgan K Jones AM. Definitions, foundations and associations of physical literacy: a systematic review. Sports Med. (2017) 47:113–26. doi: 10.1007/s40279-016-0560-7, PMID: 27365029 PMC5215133

[4] Cairney J Kiez T Roetert EP Kriellaars D. A 20th-century narrative on the origins of the physical literacy construct. J Teach Phys Educ. (2019) 38:79–83. doi: 10.1123/jtpe.2018-0072

[5] Whitehead M. “Physical literacy.” In *International Association of Physical Education and Sport for Girls and Women Congress*. Melbourne (1993).

[6] Whitehead M. Physical literacy throughout the lifecourse. London: Routledge (2010).

[7] Zhang C Liu Y Xu S Sum RK Ma R Zhong P. Exploring the level of physical fitness on physical activity and physical literacy among Chinese university students: a cross-sectional study. Front Psychol. (2022) 13:833461. doi: 10.3389/fpsyg.2022.833461 35369138 PMC8966725

[8] Barnett LM Jerebine A Keegan R Watson-Mackie K Arundell L Ridgers ND. Validity, reliability, and feasibility of physical literacy assessments designed for school children: a systematic review. Sports Med. (2023) 53:1905–29. doi: 10.1007/s40279-023-01867-4, PMID: 37341907 PMC10504218

[9] Allender S Cowburn G Foster C. Understanding participation in sport and physical activity among children and adults: a review of qualitative studies. Health Educ Res. (2006) 21:826–35. doi: 10.1093/her/cyl063, PMID: 16857780

[10] Ma RS Sum RK Hu YN Gao TY. Assessing factor structure of the simplified Chinese version of perceived physical literacy instrument for undergraduates in mainland China. J Exerc Sci Fit. (2020) 18:68–73. doi: 10.1016/j.jesf.2020.01.001, PMID: 31998384 PMC6965736

[11] Martins J Onofre M Mota J Murphy C Repond RM Vost H. International approaches to the definition, philosophical tenets, and core elements of physical literacy: a scoping review. Prospects. (2021) 50:13–30. doi: 10.1007/s11125-020-09466-1

[12] Lubans DR Morgan PJ Cliff DP Barnett LM Okely AD. Fundamental movement skills in children and adolescents: review of associated health benefits. Sports Med. (2010) 40:1019–35. doi: 10.2165/11536850-000000000-00000, PMID: 21058749

[13] Grauduszus M Wessely S Klaudius M Joisten C. Definitions and assessments of physical literacy among children and youth: a scoping review. BMC Public Health. (2023) 23:1746. doi: 10.1186/s12889-023-16680-x, PMID: 37679785 PMC10486121

[14] Pelletier LG Tuson KM Fortier MS Vallerand RJ Briére NM Blais MR. Toward a new measure of intrinsic motivation, extrinsic motivation, and amotivation in sports: the sport motivation scale (SMS). J Sport Exerc Psychol. (1995) 17:35–53.

[15] Edwards LC Bryant AS Keegan RJ Morgan K Cooper SM Jones AM. ‘Measuring’physical literacy and related constructs: a systematic review of empirical findings. Sports Med. (2018) 48:659–82. doi: 10.1007/s40279-017-0817-9, PMID: 29143266 PMC5808047

[16] Liu Y Chen S. Physical literacy in children and adolescents: definitions, assessments, and interventions. Eur Phys Educ Rev. (2021) 27:96–112. doi: 10.1177/1356336X20925502

[17] Young L O’Connor J Alfrey L. Physical literacy: a concept analysis. Sport Educ Soc. (2020) 25:946–59. doi: 10.1080/13573322.2019.1677586

[18] Sterdt E Liersch S Walter U. Correlates of physical activity of children and adolescents: a systematic review of reviews. Health Educ J. (2014) 73:72–89. doi: 10.1177/0017896912469578

[19] Ma RS Sum RK Li MH Huang Y Niu XL. Association between physical literacy and physical activity: a multilevel analysis study among Chinese undergraduates. Int J Environ Res Public Health. (2020) 17:7874. doi: 10.3390/ijerph17217874, PMID: 33121068 PMC7663683

[20] Wang FJ Choi SM Lu YC. The relationship between physical literacy and quality of life among university students: the role of motivation as a mediator. J Exerc Sci Fit. (2024) 22:31–8. doi: 10.1016/j.jesf.2023.10.002, PMID: 38054164 PMC10694319

[21] Choi SM Sum KWR Leung FLE Wallhead T Morgan K Milton D. Effect of sport education on students’ perceived physical literacy, motivation, and physical activity levels in university required physical education: a cluster-randomized trial. High Educ. (2021) 81:1137–55. doi: 10.1007/s10734-020-00603-5

[22] Cairney J Dudley D Kwan M Bulten R Kriellaars D. Physical literacy, physical activity and health: toward an evidence-informed conceptual model. Sports Med. (2019) 49:371–83. doi: 10.1007/s40279-019-01063-3, PMID: 30747375

[23] Chen X Cui J Zhang Y Peng W. The association between BMI and health-related physical fitness among Chinese college students: a cross-sectional study. BMC Public Health. (2020) 20:444. doi: 10.1186/s12889-020-08517-8 32248800 PMC7132965

[24] Li MH Whitehead M Green N Ren H Cheng CF Lin LL. Operationally defining physical literacy in Chinese culture: results of a meta-narrative synthesis and the panel's recommendations. J Exerc Sci Fit. (2022) 20:236–48. doi: 10.1016/j.jesf.2022.04.003, PMID: 35646130 PMC9117885

[25] Wang FJ Cheng CF Chen MY Sum KWR. Temporal precedence of physical literacy and basic psychological needs satisfaction: a cross-lagged longitudinal analysis of university students. Int J Environ Res Public Health. (2020) 17:4615. doi: 10.3390/ijerph17124615, PMID: 32604980 PMC7345862

[26] Chen SP. Measurement tools and applications in physical exercise research. Xi’an, China: Xi'an Jiaotong University Press (2010).

[27] Wang S Liu Y Gu C. Mechanisms of amateur sports team cohesion on members' exercise adherence: a moderated two-level mediation model. J Wuhan Inst Phys Educ. (2016) 50:73–80. doi: 10.3969/j.issn.1000-520X.2016.03.012

[28] Ma J Wang K Tylka TL. Psychometric properties of a mandarin Chinese version of the body appreciation Scale-2 with residents from Chinese mainland. Body Image. (2022) 42:110–9. doi: 10.1016/j.bodyim.2022.05.007, PMID: 35691102

[29] Wang DD. Reliability and validity test of the brief grit scale in Chinese college and middle school students. Wuhan: Wuhan Institute of Physical Education (2016).

[30] Liang DQ. The stress level of college students and its relationship with physical exercise. Chin J Ment Health. (1994) 8:5–6.

[31] Ministry of Education of the People’s Republic of China. Notice of the Ministry of Education on the issuance of the "National Student Physical Health Standard (revised in 2014)" [EB/OL]. (2024). Available online at: http://www.moe.gov.cn/s78/A17/twys_left/moe_938/moe_792/s3273/201407/t20140708_171692.html (Accessed November 07, 2024).

[32] Xing Z Huang L. Study of applying to citizens in China on Ryff’s psychological well-being scales. Health Psychol J. (2004) 12:231–3. doi: 10.3969/j.issn.1005-1252.2004.03.038

[33] Yu XN Zhang JX. Factor analysis and psychometric evaluation of the Connor-Davidson resilience scale (CD-RISC) with Chinese people. Soc Behav Pers. (2007) 35:19–30. doi: 10.2224/sbp.2007.35.1.19

[34] Rojas-Valverde D Pino-Ortega J Gómez-Carmona CD Rico-González M. A systematic review of methods and criteria standard proposal for the use of principal component analysis in team’s sports science. Int J Environ Res Public Health. (2020) 17:8712. doi: 10.3390/ijerph17238712, PMID: 33255212 PMC7727687

[35] Luo L Song N Huang J Zou X Yuan J Li C. Validity evaluation of the college student physical literacy questionnaire. Front Public Health. (2022) 10:856659. doi: 10.3389/fpubh.2022.856659, PMID: 35692349 PMC9178231

[36] Ma R Liu T Raymond Sum KW Gao T Li M Choi SM. Relationship among physical literacy, mental health, and resilience in college students. Front Psychol. (2021) 12:767804. doi: 10.3389/fpsyt.2021.767804, PMID: 34966305 PMC8710533

[37] Durden-Myers E Bartle G. Physical-literacy-enriched physical education: a capabilities perspective. Child Aust. (2023) 10:1503. doi: 10.3390/children10091503, PMID: 37761464 PMC10527893

[38] Kan W Huang F Xu M Shi X Yan Z Türegün M. Exploring the mediating roles of physical literacy and mindfulness on psychological distress and life satisfaction among college students. PeerJ. (2024) 12:e17741. doi: 10.7717/peerj.17741, PMID: 39071137 PMC11283774

[39] Kwan MYW Graham JD Bedard C Bremer E Healey C Cairney J. Examining the effectiveness of a pilot physical literacy based intervention targeting first-year university students: the PLUS program. SAGE Open. (2019) 4:1–9. doi: 10.1177/2158244019850248

[40] Zhang Y Fan S Hui H Zhang N Li J Liao L. Privacy protection for open sharing of psychiatric and behavioral research data: ethical considerations and recommendations. Alpha Psychiatr. (2025) 26:38759. doi: 10.31083/AP38759, PMID: 40110382 PMC11915712

[41] Lloyd RJ Smith S Sahingil D. Physical literacy, health and interactive aging: a position paper. Front Sports Act Living. (2024) 6:1346802. doi: 10.3389/fspor.2024.1346802, PMID: 38600905 PMC11004233

[42] Gao TY Huang FH Liu T Sum RKW De Liu J Tang D. The role of physical literacy and mindfulness on health-related quality of life among college students during the COVID-19 pandemic. Sci Rep. (2024) 14:237. doi: 10.1038/s41598-023-50958-9, PMID: 38167897 PMC10761947

